# Epicardial Adiposity in Children with Obesity and Metabolic Syndrome

**Published:** 2014-07-02

**Authors:** Erdal Eren, Bulent Koca, Mehmet Ture, Bulent Guzel

**Affiliations:** 1 Pediatric Endocrinology Division; 2 Pediatric Cardiology Division, Department of Pediatrics; 3 Department of Pediatrics, Medical School, Harran University, Sanliurfa, Turkey

**Keywords:** Adipose Tissue, Insulin Resistance, Obesity, Metabolic Syndrome, Cardiovascular Diseases, Adolescence

## Abstract

***Objective:*** Obesity increases cardiac diseases by increasing tendency to atherosclerosis. Our aim was to define epicardial adipose tissue thickness, and its related factors in obese children.

***Methods:*** Total of 94 patients were divided into obesity with metabolic syndrome (MS) (n=30), obesity without MS (n=33), and control (n=31) groups. Auxological values with fasting glucose, fasting insulin, alanine transaminase, serum lipid levels, and high sensitive C-reactive protein levels were evaluated. Epicardial adipose tissue thickness, interventricular septum thickness and left ventricular mass were measured by echocardiography.

***Findings:*** Weight, body mass index, waist circumference, insulin, alanine transaminase, and high sensitive C-reactive protein values were markedly higher in obesity group when compared with controls (*P*<0.001). Epicardial adipose tissue thickness was 0.64±0.23 cm in obesity with MS; 0.60±0.20 cm in obesity without MS, and 0.27±0.12 cm in control group (*P*<0.001). Interventricular septum thickness and left ventricular mass values were markedly high in obesity without MS group (*P*<0.001 and *P*=0.002).

***Conclusion:*** Our study has indicated that obesity has unfavorable effects on heart starting in the adolescence.

## Introduction

Obesity is an important factor threatening the health all over the world. It is known that obesity increases cardiac diseases by increasing tendency to atherosclerosis. Cardiac diseases in the adulthood are originated from childhood period, and atherosclerosis has a long asymptomatic period in children. 

 It has been shown that intra-abdominal adiposity has a correlation with insulin resistance and metabolic syndrome (MS), and it was a risk marker for cardiovascular diseases^[^^1^^]^. In adult studies, it has been shown that epicardial adipose tissue is the marker of visceral adiposity, and it is a risk factor for cardiac diseases^[^^2^^]^. Correlation between body mass index (BMI) and epicardial adipose tissue thickness has been confirmed^[^^3^^,^^4^^]^. It is also reported that epicardial adipose tissue thickness was correlated with visceral adiposity in children, but not a marker for MS^[^^5^^]^. In this present study, it was aimed to define whether there was any relationship between measurements of epicardial adipose tissue thickness (easily measured and a non-invasive technique) and MS as well as insulin resistance.

## Subjects and Methods

**Study participants: **The study was performed on patients, who applied to outpatient clinics of pediatric endocrinology and pediatric cardiology divisions. According to age and gender, patients with BMI over 95 percentile were accepted as obese^[^^6^^]^. Patients with three or more of parameters like abdominal obesity, hypertension, hyperglycemia, high triglyceride and low high density lipoprotein levels were defined as MS^[^^7^^]^. Auxological data of participants like height, weight, BMI, and waist circumference were recorded. Standard deviation score (SDS) of height, weight, and BMI were calculated^[^^8^^]^. Age and gender matched patients, who applied to outpatient clinic of pediatric cardiology with chest pain or murmur, and in whom pathology was not defined, were taken into the control group. All control cases were healthy. Subjects with systemic diseases, syndromic diseases, and chronic diseases were excluded from the study.

**Blood samples: **Fasting glucose, fasting insulin, alanine transaminase, plasma triglycerides, total cholesterol, low density lipoprotein, high density lipoprotein, high sensitive C-reactive protein levels of cases were measured in the blood samples. The homeostasis model assessment-estimated insulin resistance (HOMA-IR) index (glucose×insulin/405) was used for the detection of insulin resistance. Cases with HOMA-IR value above 3.16 were accepted as cases with insulin resistance^[^^9^^]^. 

**Measurement of lipid profiles and high sensitive C-reactive protein: **Plasma triglycerides, total cholesterol, low density lipoprotein, and high density lipoprotein were measured by an automated chemistry analyzer (Aeroset, Abbott, USA) using Abbott commercial kits. Serum high sensitive C-reactive protein level was measured using an available commercial kit (Roche).

**Fig. 1:**
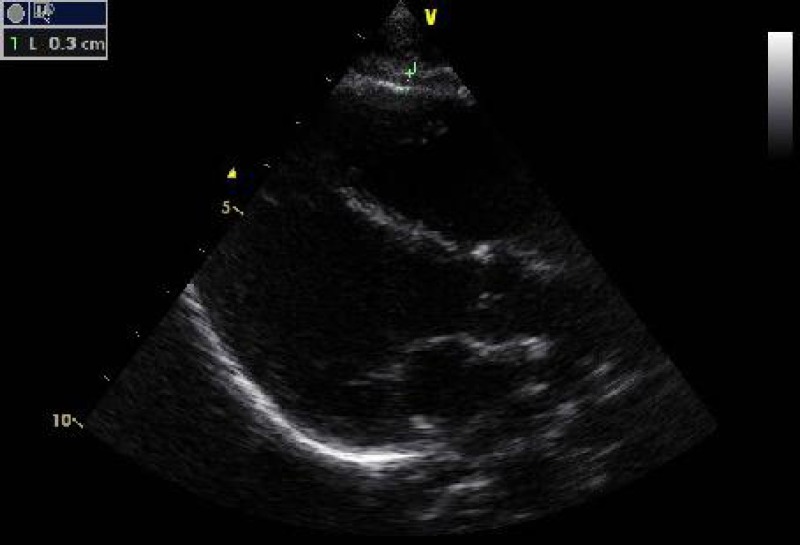
Echocardiographic measurement of epicardial adipose tissue (EAT)(plus signs indicate epicardial area)

**Echocardiographic measurements: **Echocardiographic examination was performed using a General Electric Medical Systems, USA Vivid S6 device with 4-MHz phase transducer. Examinations have been made in left lateral position on standard parasternal long axis and apical four chamber views. An electrocardiogram was simultaneously recorded in all subjects. Interventricular septum thickness was measured from the two dimensional targeted M-mode echocardiographic tracings in the parasternal long axis. The left ventricular mass was calculated by the formula (LVM=[0.8×{1.04x (IVSd+LVEDd+ LVPWd)3–LVEDd3}]+0.6)^[^^10^^]^. Epicardial adipose tissue was measured in two-dimensional echocardiography as an echo-free space over the pericardial layers and its thickness was measured on the free wall of the right ventricle, perpendicular to the wall, from parasternal long-axis view at end-diastole for three cardiac cycles (Fig. 1)^[^^11^^]^.

**Statistical Analysis: **Data were analyzed using SPSS (Statistical Package for the Social Sciences, version 11.5 for Windows, SPSS® Inc, Chicago, IL). Distribution of parametric variables was assessed with one-sample Kolmogorov–Smirnov test and all parametric variables were not distributed normally. The results were presented as mean±standard deviation. Continuous variables were compared with independent sample t-test or the Mann-Whitney U test for two groups. Kruskal-Wallis test was used to determine for differences between three groups. A two-tailed *P* value less than 0.05 was considered statistically significant.

## Findings

In the study, 94 cases (50 females and 44 males) were enrolled. Of them, 30 were in obesity with MS (15F and 15M); 33 were in obesity without MS (16F and 17M); and 31 (19F and 12M) were in control groups. Mean ages in obesity with MS, obesity without MS and control groups were 12.97±1.80 years, 12.93±1.79 years, and 13.08±1.78 years, respectively.

**Table 1:** Age, gender and auxological data of cases

	**Obesity with MS ** **(n=30)**	**Obesity without MS ** **(n=33)**	**Controls ** **(n=31)**	***P. *** **value**
**Gender**	15F/15M	16F/17M	19F/12M	0.752
**Age (years)**	12.97(1.80)	12.93 (1.79)	13.08 (1.78)	0.640
**Height (cm)**	156.60(9.72)	156.52 (9.47)	155.06 (9.20)	0.324
**Height SDS**	0.19(0.99)	0.10 (-1.10)	-0.80 (0.91)	0.212
**Weight (kg)**	72.51(18.84)	72.04 (17.28)	44.63 (8.79)	<0.001^a^,^b^
**Weight SDS**	2.14(0.58)	2.12(0.67)	-0.07 (0.91)	<0.001^a^,^b^
**Body mass index (%)**	29.08(4.83)	28.97 (4.03)	18.67 (4.17)	<0.001^a^,^b^
**Body mass index SDS**	2.42(0.31)	2.33 (0.33)	0.12 (0.81)	<0.001^a^,^b^
**Waist circumference (cm)**	91.06(12.04)	90.1 (10.43)	66.5 (8.99)	<0.001^a^,^b^

MS; Metabolic syndrome, SDS; Standard deviation score,

*P* Kruskal Wallis test,

a Difference between obesity with MS and control groups,

b Difference between obesity without MS and control groups

c Difference between obesity groups

**Fig. 2:**
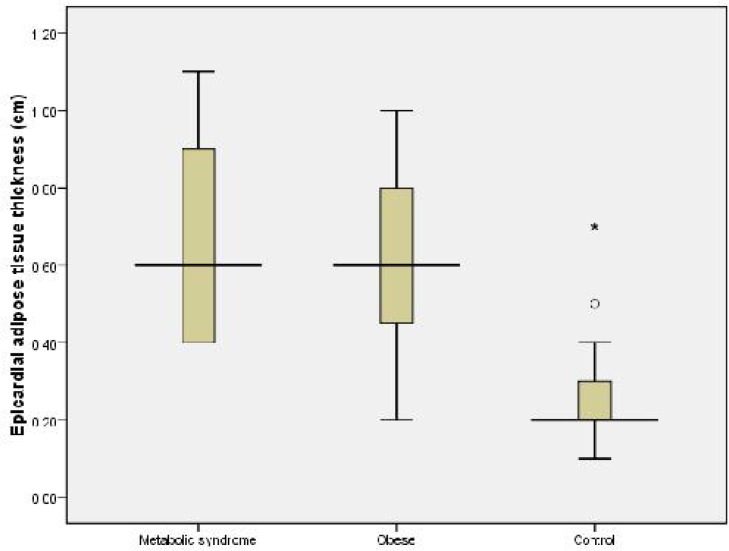
Changes in epicardial adipose tissue thickness according to groups

There was no difference in mean age, gender, height and height SDS between the groups. Mean weight, weight SDS, BMI, BMI SDS, and waist circumference were markedly higher in obesity groups when compared with controls (*P*<0.001, Table 1). While there was no difference in mean fasting glucose levels between the groups, fasting glucose and HOMA-IR values were higher in obesity groups when compared with control group. Mean fasting insulin and HOMA-IR value were statistically significantly higher in obesity with MS group when compared with the obesity without MS group (*P*<0.001). Mean alanine transaminase, high sensitive C-reactive protein level, interventricular septum thickness and left ventricular mass were statistically higher in obesity groups when compared with the controls. Mean epicardial adipose tissue thickness in obesity with MS, obesity without MS, and control groups were 0.64±0.23 cm, 0.60±0.20 cm, and 0.27±0.12 cm, respectively (*P*<0.001). There was no significant difference in epicardial adipose tissue thickness between obesity groups (Fig 2). 

 Laboratory and cardiac evaluation are shown in Table 2. When all cases were evaluated, strong positive correlations were detected between epicardial adipose tissue thickness, and weight, weight SDS, BMI, BMI SDS, waist circumference, interventricular septum thickness, and left ventricular mass (*P*<0.01, Table 3). No correlation was detected in epicardial adipose tissue thickness and fasting glucose, insulin, alanine transaminase, high sensitive C-reactive protein, interventricular septum thickness, and left ventricular mass between cases obesity with and without MS (*P*>0.05). 

**Table 2:** Laboratory and echocardiographic data of case groups

**Variable**	**Obesity with MS ** **(n=30)**	**Obesity without ** **MS (n=33)**	**Controls ** **(n=31) **	***P. *** **value**
**Fasting glucose (mg/dl)**	94,58 (12.87)	90.41 (8.10)	89.64 (4.85)	0.151
**Fasting insulin (mU/ml)**	25.36 (22.22)	10.76 (5.21)	6.68 (3.35)	<0.001^a^,^b^,^c^
**HOMA-IR **	6.69 (7.50)	2.53 (1.36)	1.53 (0.75)	<0.001^a^,^b^,^c^
**Alanine transaminase (IU/ml)**	37.91 (22.73)	23.83 (11.36)	14.25 (5.88)	<0.001^a^,^b^
**Total Cholesterol (mg/dl)**	185.42 (40.48)	176.18 (71.04)	150.65 (16.79)	0.048^a^
**Low-density lipoprotein (mg/dl)**	100.64 (33.33)	109.11 (69.43)	90.25 (18.45)	0.354
**High-density lipoprotein (mg/dl)**	37.10 (5.52)	45.37 (9.74)	43.58 (9.43)	0.010^a^,^c^
**Triglycerids (mg/dl)**	204.68 (71.46)	104.80 (41.29)	92.13 (37.86)	<0.001^a^,^c^
**High sensitive CRP (mg/l) **	0.57 (0.37)	0.54 (0.61)	0.19 (0.26)	<0.001^a^,^b^
**Epicardial adipose tissue thickness (cm)**	0.64 (0.23)	0.60 (0.20)	0.27 (0.12)	<0.001^a^,^b^
**IVS (mm)**	9.03 (1.73)	8.84 (1.46)	7.48 (1.12)	<0.001^a^,^b^
**Left ventricular mass (g)**	135.50 (62.75)	135.87 (43.31)	96.20 (32.01)	0.002^a^,^b^

MS; Metabolic syndrome, SDS; Standard deviation score, CRP; C-reactive protein, IVS; Diastolic interventricular septum thickness, HOMA-IR: Homeostasis model assessment-estimated insulin resistance

*P* Kruskal Wallis test,

a Difference between obesity with MS and control groups,

b Difference between obesity without MS and control groups

c Difference between obesity groups

When cases with obesity were evaluated according to presence of insulin resistance (when HOMA-IR threshold value was defined as 3.16), waist circumference was markedly increased in the group with insulin resistance (*P*<0.05), whereas no difference was defined in weight SDS, BMI SDS, epicardial adipose tissue thickness, alanine transaminase, high sensitive C-reactive protein, interventricular septum thickness and left ventricular mass (Table 4).

## Discussion

Intra-abdominal and epicardial adipose tissues are originated from brown adipose tissue during embryogenesis, and differentiated into white adipose tissue^[^^12^^]^. Adipose tissue is an active organ. Marked inflammatory response is observed if visceral adipose tissue has been increased. Studies indicated that epicardial adipose tissue thickness had inflammatory activity potential as the other adipose tissue storages, as well as more proinflammatory cytokines like IL-1b, IL-6, and TNF alpha than the subcutaneous adipose tissue^[^^13^^]^. In our study, high sensitive C-reactive protein levels, the marker of inflammatory response, were increased markedly in obesity and MS groups. However, there was no correlation between increased high sensitive C-reactive protein and increased epicardial adipose tissue thickness. Unfavorable impacts of abdominal adiposity on cardiovascular system have not only been observed in adults, but in children and adolescents as well. It has been reported in studies that waist circumference values indicating abdominal adiposity were more significant than BMI values^[^^14^^]^.

**Table 3:** Correlation between epicardial adiposity, left ventricle parameters and auxological data in all cases

**Variable**	**Epicardial adipose tissue ** **thickness, r**	**IVSD, r**	**LVM, r**
**Weight (kg)**	0.504*	0.597*	0.710*
**Weight SDS**	0.593*	0.507*	0.548*
**Body mass index (%)**	0.605*	0.493*	0.507*
**Body mass index SDS**	0.627*	0.465*	0.459*
**Waist circumference (cm)**	0.585*	0.581*	0.594*
**Epicardial adipose tissue thickness (cm)**		0.377*	0.351*
**IVS (mm)**			0.802*

* *P*<0.01; IVS; Diastolic interventricular septal thickness, LVM; Left ventricular mass; SDS; Standard deviation score

**Table 4:** Evaluation of all cases with obesity and metabolic syndrome according to presence of insulin resistance

**Variable**	**IR (+) (n=26)**	**IR (-) (n=37)**	***P. *** **value** *
**Weight SDS**	2.01 (0.58)	1.76 (0.64)	0.1
**Body mass index SDS**	2.04 (0.33)	1.91 (0.31)	0.1
**Waist circumference (cm)**	94.4 (11.7)	88.3 (10.15)	0.04
**Epicardial adipose tissue thickness (cm)**	0.61 (0.22)	0.63 (0.21)	0.7
**Alanine transaminase (IU/ml)**	36.05 (24.71)	24.86 (10.99)	0.08
**High sensitive C-reactive protein (mg/l) **	0.56 (0.34)	0.56 (0.38)	1
**Diastolic interventricular septum thickness (mm)**	8.96 (1.66)	8.91 (1.55)	0.9
**Left ventricular mass (g)**	139.47 (60.74)	133.03 (50.81)	0.6

* *P.* value Mann Whitney U test; IR; Insulin resistance (Homeostasis model assessment- insulin resistance >3.16), SDS; Standard deviation score

Similarly, a strong correlation was detected between epicardial adipose tissue thickness and waist circumference^[^^15^^]^. In our study, a strong correlation was detected between epicardial adipose tissue thickness and weight as well as waist circumference. Epicardial adipose tissue, which is a part of visceral adiposity, accumulates all over the heart especially around coronary arteries. Framingham heart study supported that pericardial adiposity increased the risk of coronary artery disease, and it is related to myocardial infarction^[^^16^^]^. It was defined that mean epicardial adipose tissue thickness was higher in patients with unstable ischemic cardiac disease^[^^17^^]^. Moreover, the correlation between epicardial adipose tissue thickness and cardiac arrhythmia was shown^[^^18^^]^. 

 Optimal cut-off for epicardial adipose tissue thickness associated obesity was found 0.36 cm (90% sensitivity and 87% specificity)^[^^19^^]^. Also, epicardial fat cut-off point for insulin resistance was shown 0.41 cm with 90% sensitivity and 61% specificity^[^^4^^]^. In another study, Okyay et al^[^^20^^]^ showed a cutoff point of 0.435 cm determined MS with 61.7% sensitivity and 79.2% specificity. We did not study any epicardial fat cut-off point for obesity or MS. 

 MS, which progresses with insulin resistance, is an important factor for atherosclerosis and cardiac disease. Low high density lipoprotein, one of MS criteria, indicates that there is decrease in an important antioxidant system, and a tendency for atherosclerosis^[^^21^^]^. The correlation between epicardial adipose tissue thickness and obesity, and MS was studied especially in adults. In a study, a strong correlation was defined between epicardial adipose tissue thickness, pericoronary adipose tissue thickness and MS, it was reported that they might be markers for MS^[^^22^^]^. Mazur et al^[^^5^^]^ evaluated 52 obese children with the mean age of 11.6 years, and they emphasized that there was no difference in epicardial adipose tissue thickness between obese cases with and without MS. We found that there was no difference in epicardial adipose tissue thickness between MS and obesity groups. 

 Manco et al^[^^23^^]^ evaluated epicardial adipose tissues of 30 obese children with mean age of 11.2 years by the magnetic resonance imaging technique. They reported that epicardial adipose tissue thickness was statistically significantly increased in cases with insulin resistance (HOMA-IR>2.5) in the group, and this condition caused cardiovascular risk. In our study, HOMA-IR threshold value was accepted as 3.16, which was more significant in children; no correlation was detected with epicardial adipose tissue thickness. Abacı et al^[^^4^^]^ reported that there was no correlation between epicardial adipose tissue thickness and insulin resistance. We found no positive correlation between epicardial adipose tissue thickness and fasting glucose, insulin level, and HOMA-IR in obesity groups. Moreover, epicardial adipose tissue thickness was not statistically different in IR group. In another study performed on 25 obese subjects and 24 controls with mean age of 13.0 years, marked correlation was reported between epicardial adipose tissue thickness and triglycerides, uric acid, and alanine transaminase^[^^24^^]^. In our study, no correlation was found between epicardial adipose tissue thickness and alanine transaminase, triglyceride in MS and obesity groups.

 The correlation between obesity and left cardiac function disorder was defined in adults, and left ventricular diastolic filling defect abnormalities were also shown^[^^25^^]^. In adults, it was shown that increased left ventricular mass related to obesity, left ventricle hypertrophy, and diastolic function disorders were recovered after weight loss^[^^26^^]^. In another study, it was demonstrated that systolic and diastolic functions of both ventricles were decreased in adult obese patients^[^^27^^]^. It was reported that obesity caused cardiac function disorders starting from the childhood, and left ventricle hypertrophy^[^^28^^,^^29^^]^. Ozdemir et al^[^^3^^]^ found correlation between epicardial adipose tissue thickness and left ventricular mass, left atrium diameter in obese cases. Atabek et al^[^^30^^]^ reported that left ventricular mass in MS group was markedly increased than in the group of obese children and adolescents with the mean age of 11.9 years, so it might be a marker for MS. In our study, positive correlation was detected between epicardial adipose tissue thickness and interventricular septum thickness, left ventricular mass in obese cases with and without MS. However, these two parameters were not significant in indicating MS; it did not differ in insulin resistant group.

## Conclusion

Epicardial adipose tissue thickness is correlated with BMI, and waist circumference also in children. It is detected that epicardial adipose tissue thickness was not significant in indicating MS and insulin resistance. However, interventricular septum thickness and left ventricular mass, which are markedly increased in obese patients with and without MS, have indicated that obesity affects cardiac functions starting from the childhood.
